# To Former Students

**Published:** 1880-06

**Authors:** 


To Former Students.—There are hundreds of physicians scattered over this broad land, who have been educated in part by us, and probably every one of whom would be willing to subscribe and contribute interesting cases to the pages of the *Independent Practitioner*, did they know of its existence or that we held official connection with it; we would therefore esteem it a favor if those of our old students who receive it now would send us postals containing the name and address of any of their former class-mates with whom they may be in correspondence. We endeavor to furnish a quid pro quo now, to all our readers, but we want to render the Journal still more acceptable to our subscribers, and will certainly do so as our subscription list increases in numbers. If each one of our present subscribers will only send an additional name, and two dollars, they will see a marked improvement in the next volume which begins with the Number for January 1881. But many of our readers could send us a half dozen subscribers, at least, and not subject themselves to much trouble either. Let us respectfully solicit such aid in this respect as our patrons and friends may find it convenient to bestow?

